# How we read: the combined use of MRI and novel PET tracers for the characterisation and treatment planning of masses in neuro-oncology

**DOI:** 10.1186/s40644-019-0241-5

**Published:** 2019-08-19

**Authors:** Arian Lasocki, Rodney J. Hicks

**Affiliations:** 1 Department of Cancer Imaging, Peter MacCallum Cancer Centre, Grattan St, Melbourne, Victoria 3000 Australia; 2 Sir Peter MacCallum Department of Oncology, The University of Melbourne, Parkville, Australia

**Keywords:** Magnetic resonance imaging, Positron emission tomography, Neuro-oncology, neuroradiology

## Abstract

Technical advances in imaging are well demonstrated by MRI (Magnetic Resonance Imaging) and PET (Positron Emission Tomography). Excellent anatomical detail and a lack of ionising radiation make MRI the standard of care for most neuroimaging indications, and advanced sequences are providing an ever-growing ability for lesion characterisation. PET utilising the tracer fluorine-18 fluorodeoxyglucose is widely used in oncology, while newer PET tracers are able to target a growing number of metabolic pathways and cell membrane receptors. The sequential use of these modalities harnesses the strengths of both, providing complementary diagnostic and therapeutic information.

Here we outline the ways in which we use MRI and PET in a complementary manner to improve lesion characterisation in neuro-oncology. Most commonly, an abnormality is detected on either PET or MRI, and the addition of the other modality allows a more confident diagnosis and/or demonstrates additional lesions, guiding treatment decisions and, in some cases, obviating the need for biopsy. These modalities may also be combined to guide the treatment of intracranial masses for which the diagnosis is known, such as neuro-endocrine tumour metastases or meningiomas refractory to conventional therapies.

## Background

Technical advances in imaging are well demonstrated by MRI (Magnetic Resonance Imaging) and PET (Positron Emission Tomography). Excellent anatomical detail and a lack of ionising radiation make MRI the standard of care for most neuroimaging indications, while PET is widely used in oncology for diagnosis, tumour staging, post-treatment follow-up and surveillance. The most commonly utilised PET tracer, fluorine-18-fluorodeoxyglucose (FDG), has relatively limited utility as a primary diagnostic tool in neuro-oncology, however, due to high uptake in normal brain parenchyma. This limitation has been overcome by the development of newer PET tracers targeting a variety of metabolic pathways or cell membrane receptors. The sequential use of these modalities harnesses the strengths of both, providing complementary information to optimise diagnosis and treatment planning. These complementary strengths have also led to the development of combined PET-MR systems, which provide improved image coregistration [1] and a lower dose of ionising radiation compared to PET-CT (Computed Tomography), with greater patient convenience.

Here we discuss the ways in which we use MRI and PET in a complementary manner to improve lesion characterisation in neuro-oncology, with illustrative clinical examples. As the cornerstone of neuroradiology, MRI provides adequate characterisation of most intracranial lesions, and advanced sequences are further increasing the information available. In selected cases, however, the addition of PET provides complementary molecular characterisation and in certain circumstances can raise diagnostic confidence to a level that can avoid need for confirmatory biopsy. Most commonly, an abnormality is detected on either PET-CT or MRI, and the addition of the other modality allows a more confident diagnosis. These modalities may also be combined to guide the treatment of an intracranial mass for which the diagnosis is known.

### Imaging protocol

In many cases, the MRI and PET will not be reported by the same individual, thus interaction between the reporting neuroradiologist and nuclear medicine physician is important. The key factor is an understanding of how each modality may add value to the diagnostic process – in particular the specific diagnoses which may take up a given PET tracer – and this guides the subsequent imaging protocol.

The MRI protocol for further characterising an abnormality seen on PET should specifically target the differentials based on the PET appearances. Volumetric pre- and post-contrast T1-weighted imaging are important, as one of the key advantages of MRI over PET is the improved anatomical delineation that fine-slice imaging provides. As standard, we also suggest axial T2-weighted imaging, FLAIR (Fluid Attenuated Inversion Recovery), DWI (diffusion-weighted imaging) and a susceptibility-sensitive sequence such as SWI (Susceptibility-Weighted Imaging), as these can provide a confident diagnosis of pathologies such as a glioma [2, 3] or pyogenic abscess [4, 5]. Perfusion-weighted imaging and spectroscopy may be added depending on the differential diagnosis based on PET.

Determining an appropriate PET tracer for further characterising an abnormality seen on MRI depends on the possible differentials based on the MRI appearances and the differential tracer uptake of these entities. Similarly, to improve characterisation of a known entity, the tracer choice will be tailored to the clinical question. Tracer choice may also be influenced by local factors such as the presence of an on-site cyclotron, but it may be possible to replace with an equivalent tracer (for example, in the case of amino acid tracers).

## How we read MRI and PET together in neuro-oncology

### Incidental findings on PET staging studies

**Fig. 1:**
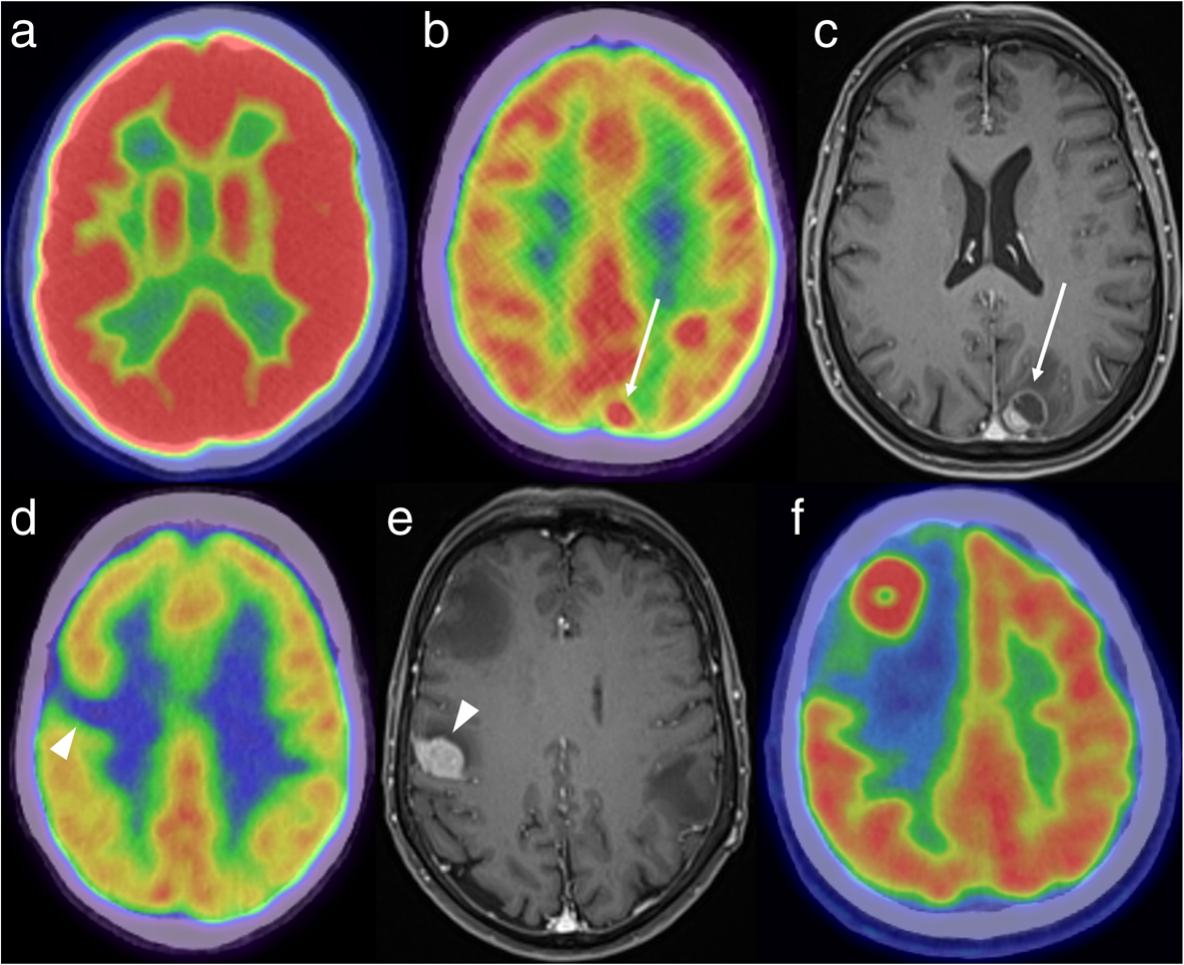
FDG-PET demonstrating normal high background uptake (**a**) - uptake is higher in the grey matter than in the white matter. A focus of high FDG uptake in the left parietal lobe (**b**, white arrow) corresponds to a mixed solid/cystic metastasis on the post-contrast MRI (**c**). An area of low uptake (**d**, white arrowhead) can also be due to a metastasis, as demonstrated on the corresponding MRI (**e**). FDG-PET in another patient (**f**) shows an FDG-avid mass in the right frontal lobe with surrounding photopaenia, consistent with oedema. Histology confirmed a solitary metastasis from a lung primary

**Fig. 2:**
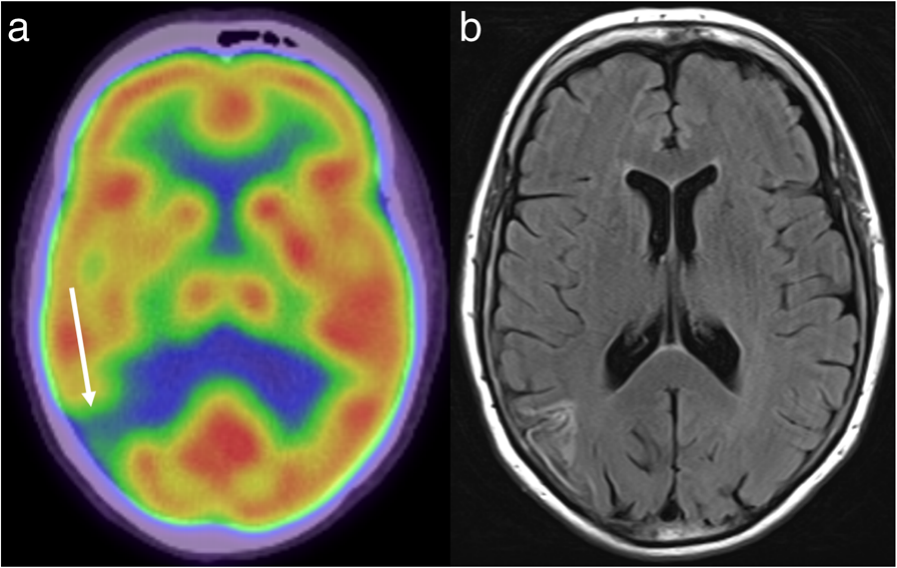
FDG-PET (**a**) in a patient with metastatic melanoma demonstrates low uptake in the right parietal lobe (arrow). The FLAIR sequence of the corresponding MRI (**b**) is consistent with a previous infarct rather than a metastasis

Despite the relative limitations of FDG-PET in the brain, the presence of an unexpected intracranial abnormality on PET is most common on FDG-PET studies, performed either for primary staging or post-treatment re-staging. The identification of an abnormality then prompts dedicated neuroimaging such as CT or MRI to characterise the abnormality, and potentially look for additional lesions below the spatial and contrast resolution of FDG-PET. The appearance of intracranial pathology on FDG-PET is somewhat dependant on its location, as normal grey matter has substantially higher background uptake than white matter. In some cases, intracranial pathology, such as a metastasis, is visualised as an area of high tracer uptake. Not uncommonly, however, metastases are masked by the high background uptake in the brain, especially when centred on the cortex. In these situations, a mass may instead be inferred by an area of relative photopaenia, reflecting the vasogenic oedema surrounding the mass. Sometimes, both an FDG-avid metastasis and the surrounding photopaenia may be visualised. These different appearances are illustrated in Fig. 1. MRI has particular value when PET demonstrates focal decreased uptake, as non-neoplastic aetiologies, such as an infarct, may also produce this appearance (Fig. 2).

**Fig. 3:**
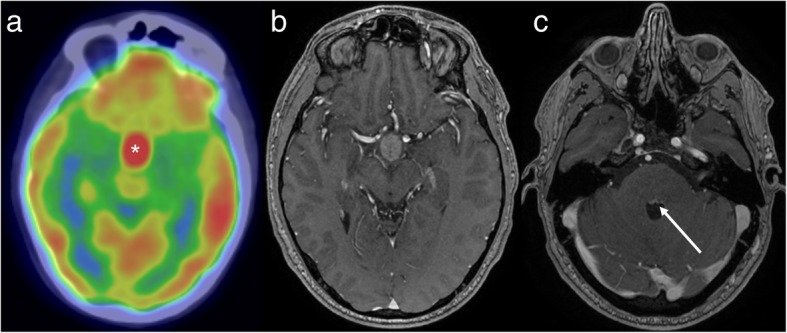
FDG-PET (**a**) in a patient with systemic lymphoma shows abnormal intracranial uptake (asterisk), consistent with secondary CNS involvement. This is localised to the hypothalamus on the post-contrast MRI (**b**). The post-contrast MRI (**c**) also demonstrates more extensive leptomeningeal disease than is appreciable on PET, including along the ependymal surface of the fourth ventricle (arrow)

MRI is also useful when PET staging for a primary extracranial lymphoma demonstrates secondary intracranial involvement. In this setting, MRI improves the anatomical localisation of disease and provides a better assessment of disease extent. There is particular value in identifying leptomeningeal disease, which is important clinically but often below the resolution of PET, especially when linear in morphology (Fig. 3). Other neoplasms such as high-grade gliomas are also typically FDG-avid [6], but are encountered much less frequently as an incidental finding.

Newer PET tracers targeting the somatostatin receptor are being increasingly used for the diagnosis and management of neuroendocrine tumours (NETs), such as those occurring in the pancreas or lungs, and paragangliomas (including phaeochromocytomas and extra-adrenal paragangliomas). The most commonly used of these agents is gallium-68 labelled 1,4,7,10-tetraazacyclododecane-N,N′,N″,N″’-tetraacetic acid (DOTA)-Tyr3-octreotate (also known as GaTate, DOTA-octreotate or DOTATATE). NETs are a heterogeneous group, varying by location of the primary and rate of proliferation. As such, they vary in their propensity to metastasise to the brain. For example, small cell lung carcinoma, the best-known high-grade neuroendocrine malignancy, is frequently associated with brain metastases [7]. In contrast, paraganglioma only rarely metastasises to the brain [8].

GaTate-PET studies performed during re-staging of a NET may demonstrate an area of unsuspected tracer uptake intracranially. The differential diagnosis is based on tumours in this location which express somatostatin receptors, the main differentials being a NET metastasis, a meningioma (as meningiomas frequently express somatostatin receptors [9] and are commonly found incidentally) and a primary intracranial neoplasm that expresses somatostatin receptors, such as a haemangioblastoma [10, 11] or esthesioneuroblastoma [12]. Gliomas variably contain somatostatin receptors and are also in the differential, though the expression of somatostatin receptors is typically lower than in meningiomas [11, 13]. In the paediatric and young adult population, the differential can be expanded to include primary embryonal tumours such as medulloblastoma [13, 14]. In general, higher grade primary brain tumours would occur rarely as an incidental finding, other than in the setting of an underlying germline mutation. In particular, von Hippel Lindau (VHL) disease can be associated with pancreatic NET, phaeochromocytoma and haemangioblastoma [15], all of which can express somatostatin receptors, as well as the well-recognised association with renal lesions, which do not.

**Fig. 4:**
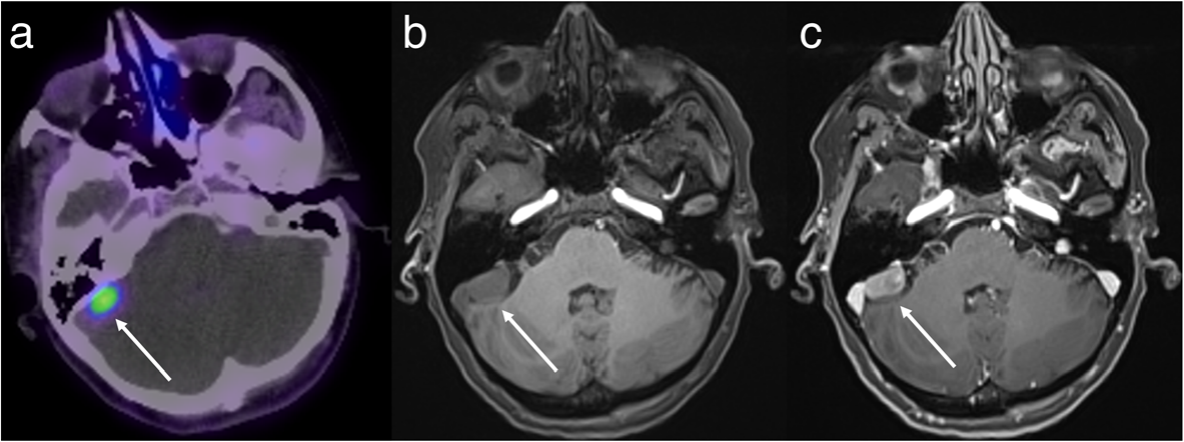
GaTate-PET (**a**) reveals a right posterior fossa mass (arrow). The pre- and post-contrast MRI images (**b** and **c**, respectively) demonstrate a homogeneously-enhancing durally-based mass, consistent with a meningioma. This diagnosis was also supported by evidence of calcification on CT (not shown)

**Fig. 5:**
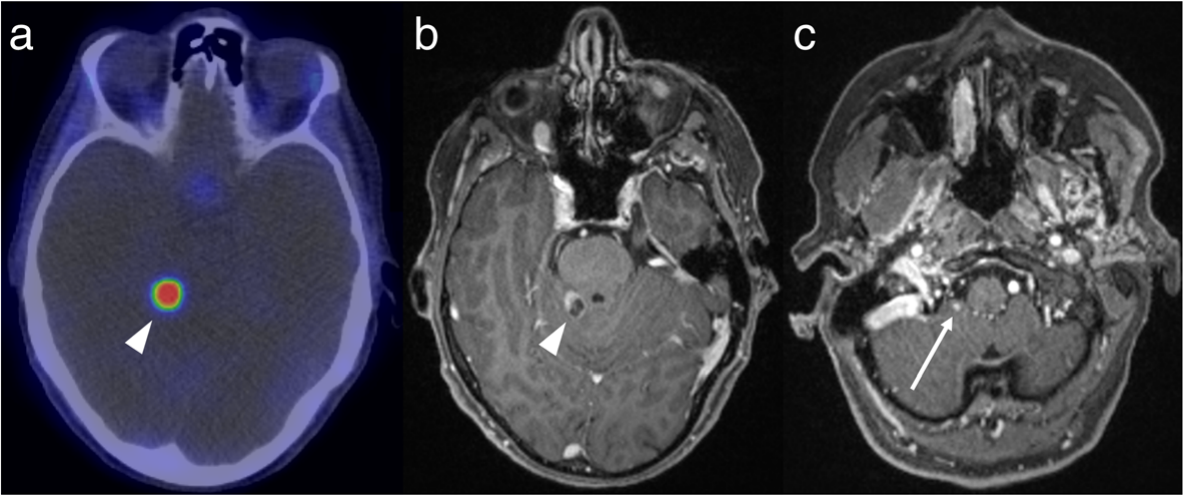
GaTate-PET (**a**) in a patient with metastatic NET identifies a lesion in the posterior fossa (arrowhead). Given the proximity to the tentorium cerebelli, both an incidental meningioma and a NET metastasis are in the differential. The mixed solid and cystic appearance on the post-contrast MRI (**b**) confirms a metastasis. MRI also demonstrates a smaller enhancing focus more inferiorly in the posterior fossa (**c**), consistent with a further NET metastasis

**Fig. 6:**
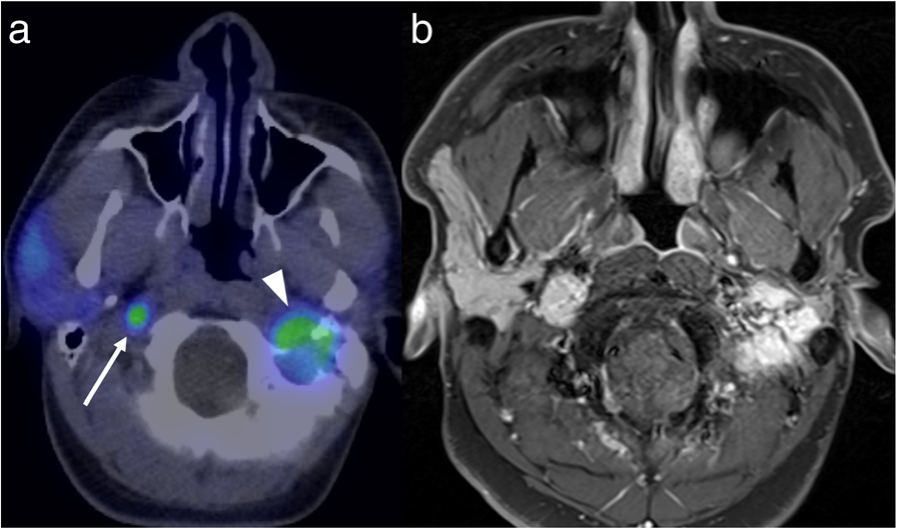
FDG-PET (**a**) performed for follow-up of a patient with a germline succinate dehydrogenase subunit B mutation and a known left glomus jugulare paraganglioma (arrowhead) demonstrates a new area of FDG-avidity just below the skull base on the right (arrow). The subsequent post-contrast MRI (**b**) supports that this is a new paraganglioma rather than a metastasis

MRI may then provide a specific diagnosis from this limited differential, for example by demonstrating the extra-axial location and dural tail of a meningioma (Fig. 4), cortical FLAIR hyperintensity in a glioma [2, 3], the characteristic cystic mass with a contrast-enhancing mural nodule in the case of a haemangioblastoma [10], the presence of additional lesions in a patient with metastatic disease (Fig. 5), or the olfactory groove epicentre of an esthesioneuroblastoma. GaTate-PET also plays an important role in the screening of patients with a genetic predisposition to NETs (Fig. 6), such as patients with germline SDH (succinate dehydrogenase) mutations (being predisposed to phaeochromocytomas and extra-adrenal paragangliomas) [16] and, as mentioned above, von Hippel-Lindau disease [15]. Indeed, the presence of additional lesions on GaTate-PET performed for follow-up of patients with a solitary neuroendocrine tumour may prompt investigation for an underlying germline mutation that was previously unsuspected [10].

**Fig. 7:**
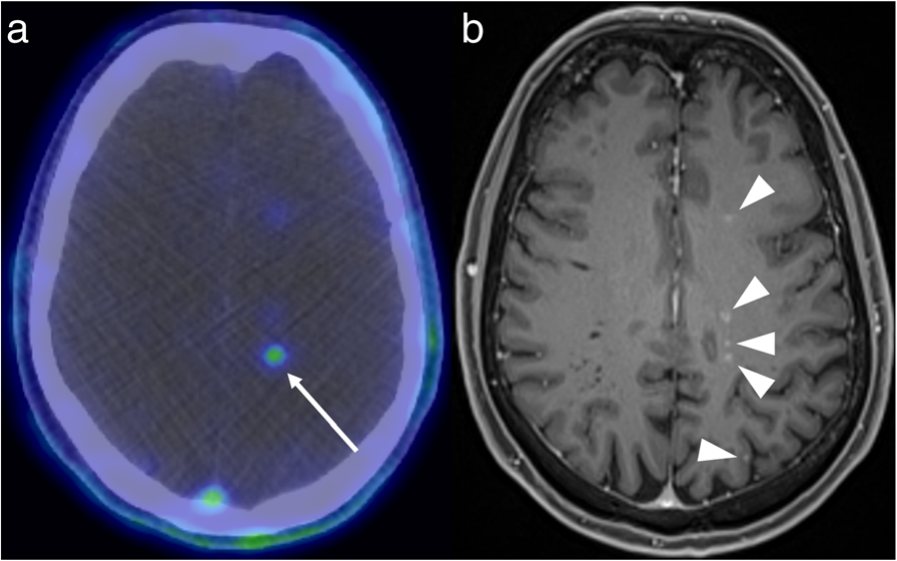
PSMA-PET (**a**) in a patient with prostate cancer shows a focus of high uptake intracranially (arrow). The post-contrast MRI (**b**), however, demonstrates much more widespread intracranial metastatic disease (arrowheads)

There has also been recent growth in the development of other targeted PET tracers. A good example is PSMA (prostate-specific membrane antigen), which has high sensitivity and specificity for the detection of prostate cancer metastases [17]. PSMA-PET may also demonstrate intracranial metastases, though this is an uncommon finding. MRI may then better demonstrate the extent of the intracranial metastatic disease, important for treatment planning. For example, surgical resection may not be feasible if MRI demonstrates more widespread metastatic disease than is visible on PET (Fig. 7).

### Further characterisation of a mass found on MRI

MRI is the standard of care for the investigation of neurological symptoms and the characterisation of an abnormality identified of other imaging modalities. It can frequently suggest a specific diagnosis, but there remain cases in which the diagnosis remains uncertain. Frequently, neurosurgery is warranted for both diagnosis and treatment, but if nonoperative management is being considered depending on the diagnosis, PET may allow a more confident diagnosis without the need for craniotomy. This is particularly relevant given the growth of non-operative therapeutic techniques such as stereotactic radiosurgery or the use of systemic radionuclide therapies.

There has been a growth in the use of PET in neuroimaging due to the development of amino acid tracers such as FET (fluorine-18-fluoroethyl-L-tyrosine), MET (carbon-11-methyl-L-methionine) and FDOPA (fluorine-18-fluoro-L-dihydroxyphenylalanine). In contrast to FDG, these tracers do not exhibit significant uptake in normal brain parenchyma, which would otherwise limit detection and characterisation of the lesion. Amino acid PET can differentiate between intracranial neoplasms (including glioma, lymphoma and metastasis), which typically demonstrate high tracer uptake, and non-neoplastic aetiologies [6, 18]. This information, combined with conventional and advanced MRI sequences, may provide a more confident diagnosis. For example, a non-FET-avid intracranial mass has a limited differential of non-malignant conditions, including abscess [19] and tumefactive demyelination [20]. Most grade III and IV gliomas (> 95%) [21] and grade II oligodendrogliomas demonstrate high tracer uptake [18], but uptake is more variable in grade I and II astrocytomas, with approximately 30% exhibiting low uptake [18].

**Fig. 8:**
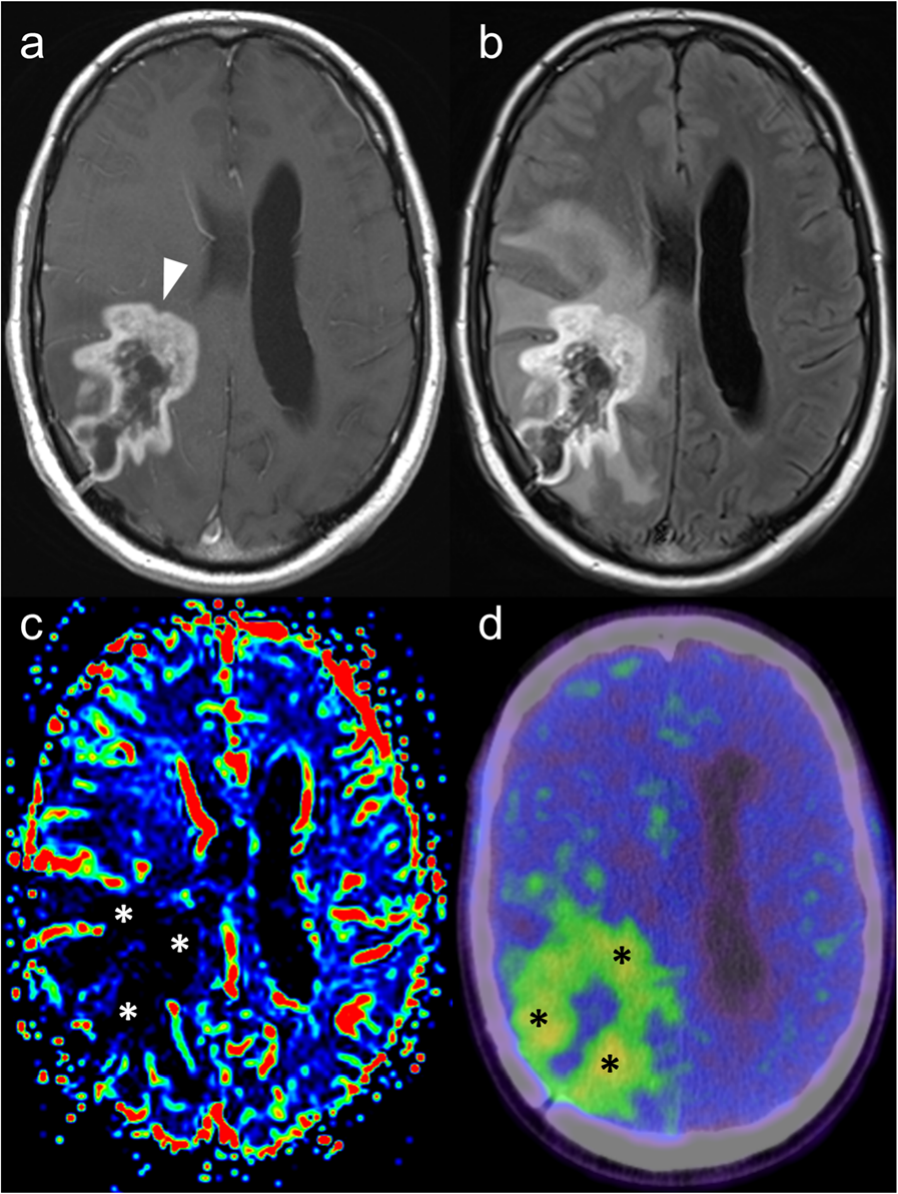
Post-contrast T1-weighted (**a**) and FLAIR (**b**) MRI images demonstrate an irregular peripherally-enhancing lesion in a patient with a known right temporo-parietal glioblastoma treated with temozolamide and radiotherapy. Given an absence of elevated cerebral blood volume on dynamic susceptibility contrast MRI perfusion (**c**), the possibility of pseudoprogression was raised. FET-PET (**d**) showed prominent tracer uptake, however, consistent with true tumour progression, which was confirmed histologically

Amino acid PET has a variety of possible uses in the context of glioma, extensively outlined in a recent consensus statement [18]. At diagnosis, it can aid surgical planning, by targeting the highest uptake component for biopsy [18]. There is also a role for delineation of tumour extent prior to surgery or radiotherapy [18], which is especially relevant given recent findings that extending resection to the non-enhancing component of a glioma provides a survival benefit [22–24]. At follow-up, amino acid PET can help differentiate between pseudo-progression and true progression (Fig. 8), and between response and pseudo-response in patients treated with anti-antiogenic agents [18] – both scenarios being challenging for MRI even when advanced techniques are utilised. Similarly, in patients with metastatic disease treated with stereotactic radiosurgery, FET-PET is useful in distinguishing between recurrent tumour and radiation necrosis, based on differences in the tumour-to-brain uptake ratios and time-activity curves [25].

**Fig. 9:**
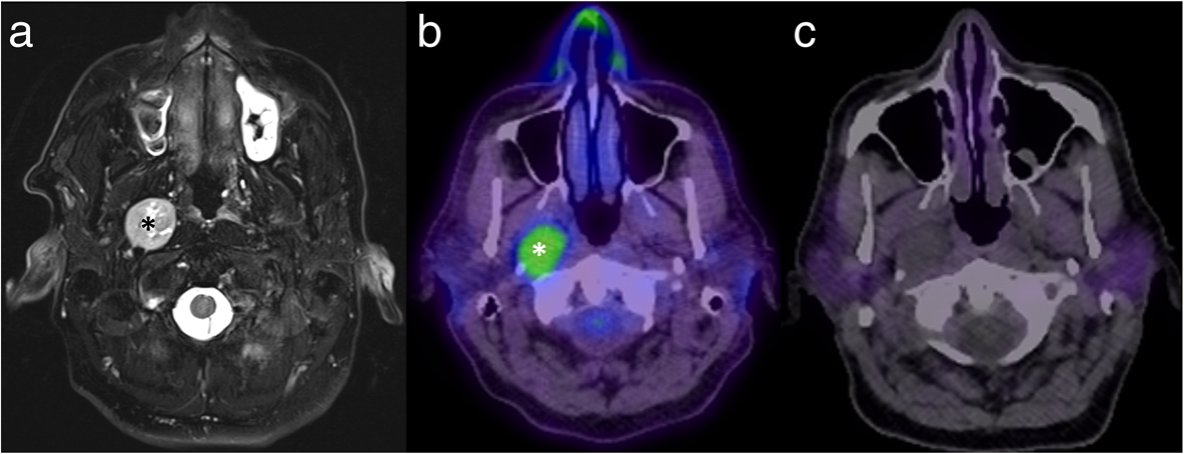
Axial T2 with fat saturation MRI (**a**) shows a mass in the right carotid space (asterisk), slowly enlarging on serial imaging (thus going against a metastasis). There is high uptake on FDG-PET (**b**), but no uptake on GaTate-PET (**c**), most consistent with a nerve sheath tumour (confirmed histologically)

Targeted PET tracers have a potential role in the characterisation of an undifferentiated mass and the choice of tracer – and thus the potential added benefit of PET – depend on the differential diagnosis for the given lesion. For example, in the context of a mass around the skull base or within the carotid space, the main differentials to consider include a metastasis, paraganglioma and nerve sheath tumour. In this setting, the targeted nature of GaTate-PET allows the diagnosis of a paraganglioma to be either confidently diagnosed or excluded, without the risks and morbidity of open biopsy (Fig. 9). In a series examining 17 patients with metastatic phaeochromocytoma/paranglioma related to SDH type B mutations, GaTate-PET detected 285 (98.6%) of 289 suspected metastases – higher than other functional imaging techniques and CT/MRI [26]. The lack of an optimal gold standard limits the assessment of specificity in such studies, however, as histological confirmation of small lesions not detected on other modalities is uncommon.

**Fig. 10:**
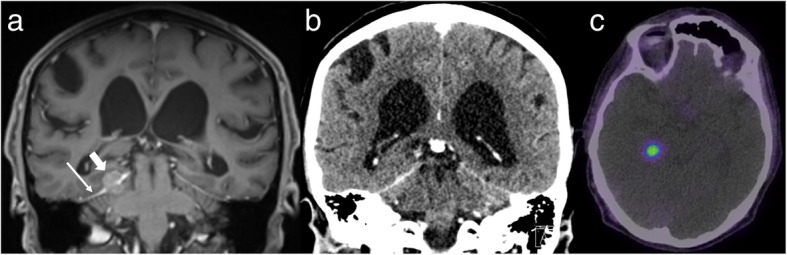
Routine post-contrast MRI surveillance (**a**) for a patient with metastatic melanoma demonstrates a durally-based mass related to the right side of the tentorium cerebelli, best seen in the coronal plane (short arrow). The appearances are suggestive of a meningioma, but the lesion was much smaller on a CT performed only 8 months earlier (**b**), raising concern for a metastasis. Further characterisation with GaTate-PET (**c**) demonstrates high uptake, confirming the diagnosis of a meningioma rather than a metastasis

Another relatively common clinical situation is differentiating between a meningioma, which is commonly found incidentally, and a dural metastasis from a non-NET primary. Often, a followup MRI to demonstrate stability of a durally-based mass will be adequate. This may not be practical in the setting of known metastatic malignancy, however, in particular with primaries known to be associated with dural metastases, such as breast or prostate [27]. Similarly, the rate of growth may occasionally be greater than can be comfortably attributed to a meningioma. In such cases, GaTate-PET can be a useful problem-solver, with the presence of GaTate-avidity being strong evidence of a meningioma (Fig. 10), while a metastasis is the likely diagnosis otherwise. While a previously-unidentified neuroendocrine component to the metastatic disease could provide an exception, this should be readily identifiable by the presence of GaTate uptake in other metastases. Non-GaTate-avid meningiomas are rare – in a series of 192 suspected meningiomas identified on GaTate-PET and/or MRI, only two identified by MRI demonstrated no GaTate uptake, and there was no histological correlation to confirm that these were indeed false negatives on GaTate-PET [28]. The main limitation of GaTate-PET in this setting is a parasellar location, due to difficulty delineating uptake from that occurring normally in the pituitary gland [29].

Other targeted tracers, such as PSMA, can also be used in this way. For example, in a patient with a history of prostate cancer presenting with a durally-based mass, PSMA-PET could differentiate between dural metastatic disease and an incidental meningioma. Targeted PET tracers may also be useful in patients with a history of multiple malignancies presenting with intracranial metastases, allowing the histology to be determined and appropriate therapy instituted non-invasively. As new targeted PET tracers become available, this will increase the complementary value of MRI and PET.

### Treatment planning

**Fig. 11:**
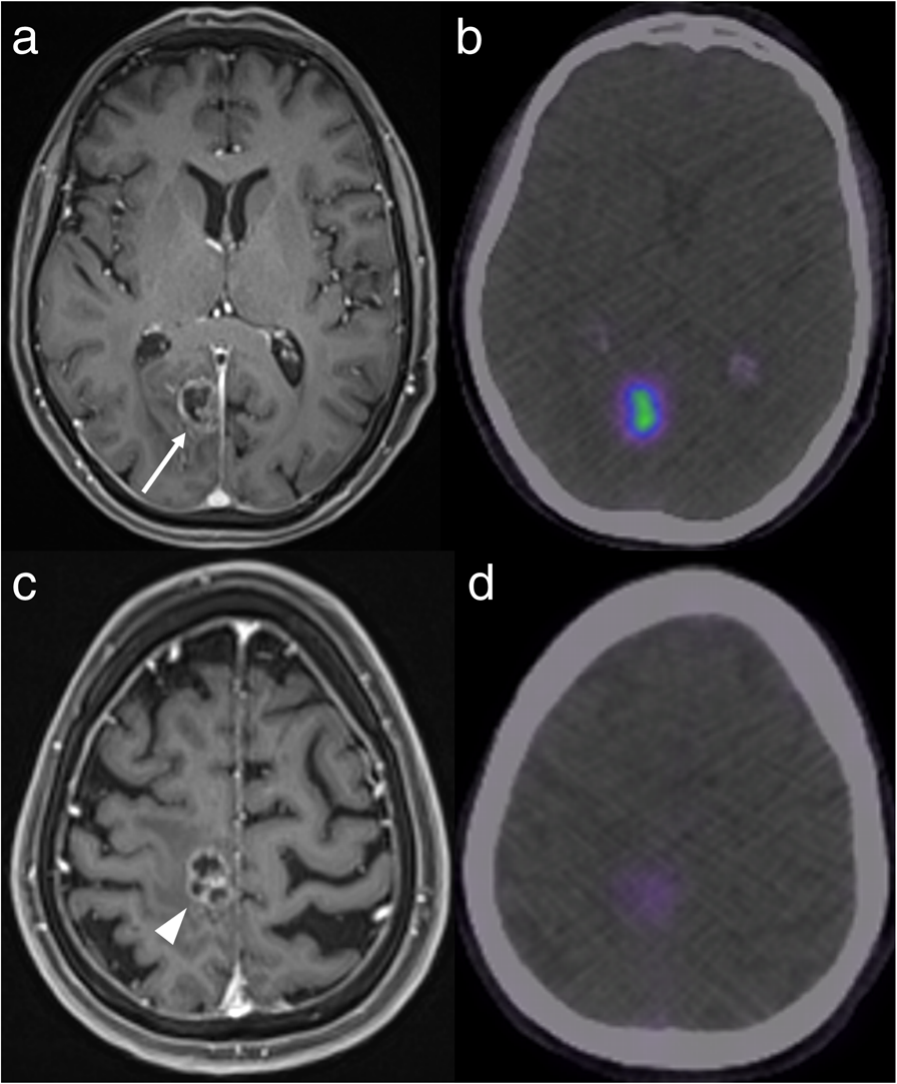
Corresponding post-contrast MRI (left) and GaTate-PET (right) images of two cerebral metastases in a patient with metastatic NET. The medial right occipital metastasis (**a**; arrow) demonstrates high GaTate uptake (**b**). If this were a solitary metastasis, a response to PRRT would be expected. The medial right pre-central gyrus metastasis (**c**; arrowhead), however, demonstrates low GaTate uptake (**d**), and is unlikely to respond to PRRT

**Fig. 12:**
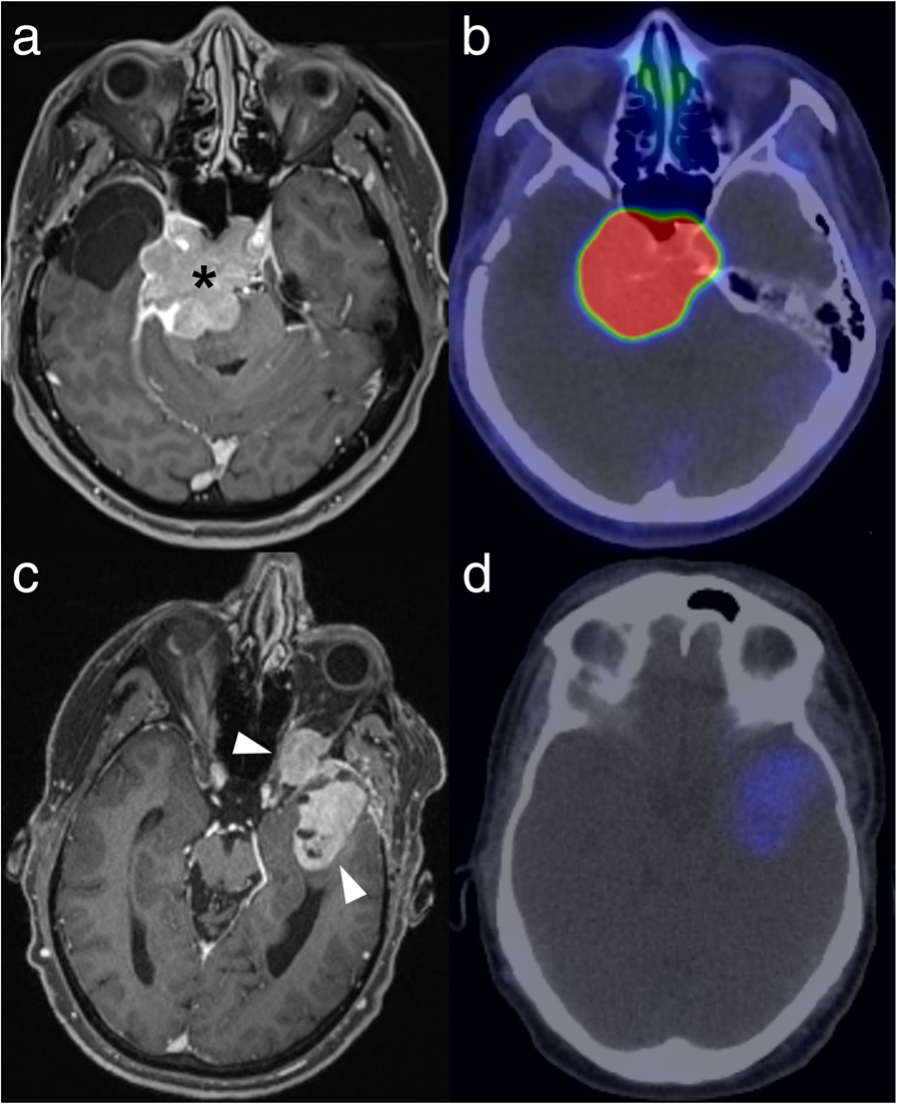
Post-contrast MRI (left) and GaTate-PET (right) images of two different patients with meningiomas refractory to conventional therapy. The skull base meningioma (**a**; asterisk) demonstrates high GaTate uptake (**b**) and may benefit from PRRT. In contrast, the left temporal meningioma extending into the orbit (**c**; arrowheads) has only low-grade GaTate uptake (**d**), thus PRRT is not warranted

**Fig. 13:**
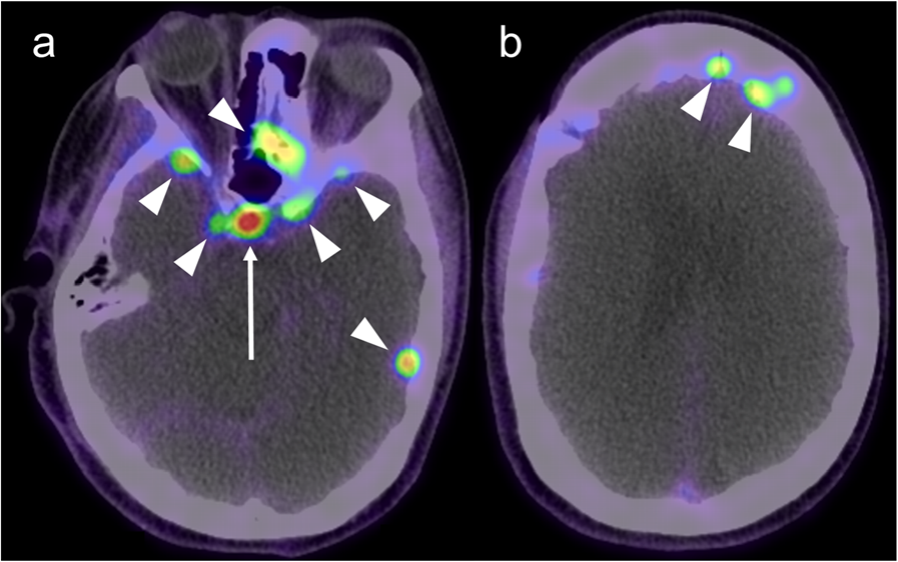
Post-contrast MRI (**a**) and GaTate-PET (**b**) in a patient with previous surgery for meningioma. A small enhancing nodule related to the falx cerebri (arrows) demonstrates GaTate-avidity, consistent with meningioma. In contrast, the more diffuse dural thickening (arrowheads) does not demonstrate GaTate uptake, and is thus consistent with post-operative change rather than en plaque meningioma

**Fig. 14:**
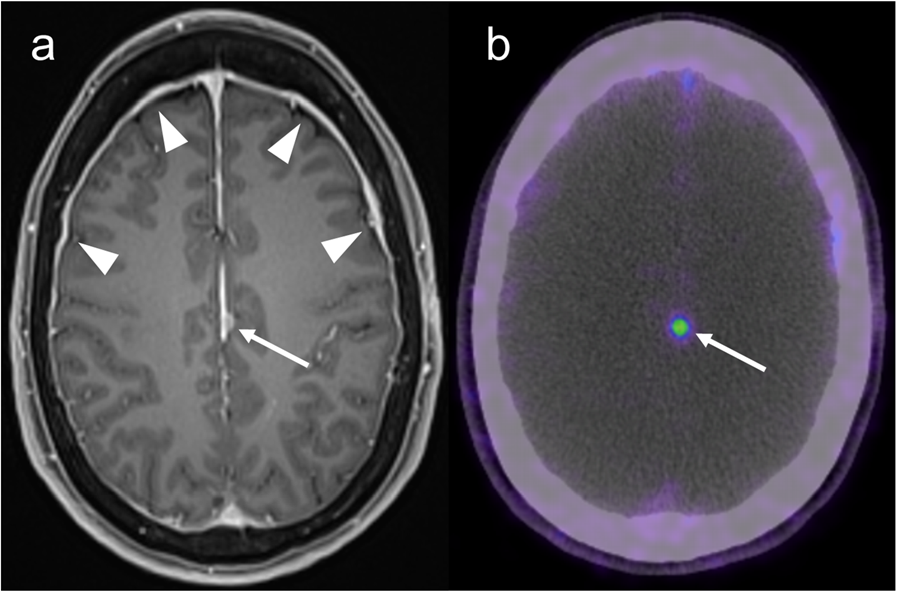
GaTate-PET demonstrating multiple scattered foci of meningioma (arrowheads). Normal GaTate uptake in the pituitary gland is noted (arrow)

GaTate-PET also has value for treatment planning when the diagnosis is known, which is particularly relevant to the growing field of theranostics, with PET tracers being used for both diagnosis and treatment (peptide receptor radionuclide therapy, or PRRT) [30]. For example, DOTATATE can be chelated with lutetium-177 or yttrium-90 to provide radiotherapy targeted to somatostatin receptor-expressing lesions [30]. GaTate-PET is first used to predict the response to PRRT by assessing the degree of tracer uptake. Uptake is measured on the Krenning scale: 0 = no uptake; 1 = very low uptake; 2 = uptake less than or equal to that of liver; 3 = greater than liver; 4 = greater than spleen [30, 31]. If all metastases demonstrate uptake greater than liver (Krenning 3), there is likely to be a better response to PRRT. In contrast, however, PRRT is unlikely to provide improvement if uptake is Krenning 2 or less in at least one of the metastases [31] (Fig. 11). Similar principles can also be used to plan PRRT for other somatostatin receptor-expressing tumours refractory to conventional therapies, such as meningioma (Fig. 12), medulloblastoma [32] and esthesioneuroblastoma [33, 34]. There is also a role for GaTate-PET in delineating the extent of meningiomas, in particular when planning radiotherapy [28]. This is particularly useful when accurate delineation is challenging on MRI alone, for example after surgery (Fig. 13) or in the setting of en plaque or multiple meningiomas (Fig. 14).

## Conclusion

MRI and PET are powerful diagnostic tools, and the complementary strengths of both can be harnessed for improving diagnostic specificity and treatment planning. This is a growing field, related to the development of novel PET tracers and the increasing utilisation of simultaneous PET-MR scanners.

## Data Availability

Not applicable.

## Abbreviations

CT: Computed Tomography
FDG: Fluorine-18 fluorodeoxyglucose
FDOPA: Fluorine-18-fluoro-L-dihydroxyphenylalanine
FET: Fluorine-18-fluoroethyl-L-tyrosine
GaTate: Gallium-68 labelled 1,4,7,10-tetraazacyclododecane-N,N′,N″,N″’-tetraacetic acid (DOTA)-Tyr3-octreotate
MET: Carbon-11-methyl-L-methionine
MRI: Magnetic Resonance Imaging
NET: Neuroendocrine tumour
PET: Positron Emission Tomography
PRRT: Peptide receptor radionuclide therapy
PSMA: Prostate-specific membrane antigen
